# Study on the Correlation Between Fermentation Microbial Communities and Flavor Compounds in Sauerkraut

**DOI:** 10.1002/fsn3.72467

**Published:** 2026-10-28

**Authors:** Xiangcheng Lu, Shiyu Guo, Hongmei Zhao, Yanjun Yang, Xiaoli Ma, Shengli Shi, Bianfang Hu, Aiguo Luo

**Affiliations:** ^1^ Department of Biological Science and Technology Jinzhong University Jinzhong China; ^2^ Shanxi Center of Technology Innovation for Compound Seasonings Jinzhong University Jinzhong China; ^3^ College of Food Science and Engineering Shanxi Agricultural University Jinzhong China; ^4^ Jinzhong Normal College Jinzhong China

**Keywords:** correlation analysis, flavor compounds, microbial community structure, sauerkraut

## Abstract

This study aimed to investigate the microbial community structure and its association with flavor compounds in mature sauerkraut fermentation. DNA high‐throughput sequencing and gas chromatography–mass spectrometry (GC–MS) were employed to analyze the microbial communities and volatile flavor compounds in sauerkraut samples on the 20th day of fermentation. A total of 104 volatile compounds were identified, including aldehydes (28.98%), alcohols (3‐methyl‐1‐butanol, 18.28%), esters (methyl thiolacetate, 3.14%), and organic acids (acetic acid, 4.28%). These compounds were associated with the characteristic sour, fruity, and grassy aroma profiles of sauerkraut. Microbial community analysis indicated that bacteria exhibited greater diversity than fungi, with Firmicutes (69.37%) and Proteobacteria (29.92%) being the dominant phyla. The genera *Enterococcus* and *Lactococcus* accounted for over 50% of the bacterial community, representing dominant taxa associated with fermentation characteristics. These microorganisms may participate in flavor development through metabolic activities related to carbohydrate utilization, amino acid metabolism, and volatile compound formation. Correlation analysis further demonstrated a significant positive relationship between *Lactococcus*, *Enterococcus*, and several acetate‐derived esters, suggesting a close statistical association between these genera and the accumulation of these compounds. This study provides further evidence for the association between microbial community structure and flavor profiles in sauerkraut, providing a scientific basis for the optimization of traditional fermented food processes and quality improvement.

## Graphical abstract

The graphical abstract illustrates the process flow of low‐temperature fermented sauerkraut, transforming fresh cabbage into the final product. The middle section presents comprehensive analyses of flavor compounds via chromatography and the microbial community composition (bacteria and fungi) through pie charts. Furthermore, the correlation heatmap on the right highlights the associations between microbial diversity and flavor compounds.

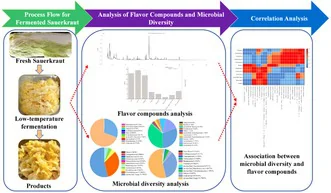

## 1 Introduction

As a traditional fermented vegetable product, sauerkraut is widely popular around the world due to its unique flavor and rich nutritional value. It is abundant in vitamin C, dietary fiber, and various trace elements, offering health benefits such as promoting digestion and enhancing immunity. According to statistics from the China National Food Industry Association, China's annual production of pickled vegetables exceeds 4 million metric tons, with the market scale continuously expanding, demonstrating significant industrial potential. The distinctive flavor and taste of sauerkraut result from the fermentation activities of microorganisms such as lactic acid bacteria under anaerobic conditions (Wook et al. 2020). Through the fermentation process, the flavor of sauerkraut is optimized, not only enhancing appetite but also improving its nutritional value (Tullio 2024).

In the microbial fermentation process of traditional fermented food such as sauerkraut, the structure of the microbial community undergoes significant dynamic changes, which are closely associated with both the safety and flavor characteristics of the final product (Cheng et al. 2022). However, many existing studies exhibit certain limitations, such as focusing on single microbial taxa while neglecting the synergistic effects of multi‐species interactions on flavor development (Jungles et al. 2025); relying heavily on targeted analysis for flavor compound identification, thereby failing to comprehensively profile volatile components; and often remaining at the level of phenomenological correlation analysis without further validation of metabolic pathways in key functional microorganisms (Wang et al. 2020). Moreover, although the microbial community structure directly influences the quality and flavor of sauerkraut (Yang et al. 2021), traditional spontaneous fermentation processes involve complex and unstable microbiota, which can lead to inconsistencies in sensory attributes and even potential safety risks. Therefore, elucidating the correlation between microbial succession and flavor formation during sauerkraut fermentation is not only of profound significance for understanding the intrinsic patterns of microbial dynamics, but also crucial for guiding industrial practices, optimizing fermentation processes, and improving product quality.

During fermentation, lactic acid bacteria metabolize sugars to produce organic acids including lactic acid and acetic acid, while also degrading macromolecules including proteins and lipids to generate flavor compounds including amino acids and free fatty acids (Guo et al. 2025). These processes collectively contribute to the characteristic sour, sweet, umami, and crisp notes of fermented sauerkraut (Lee et al. 2024). Consequently, the species composition and abundance of lactic acid bacteria directly influence both the fermentation progression and the final product quality (Liu et al. 2010; Du et al. 2014). In this study, sauerkraut was prepared from fresh sauerkraut via low‐temperature spontaneous fermentation. The correlation between the microbial community structure and flavor compounds was investigated using DNA high‐throughput sequencing and gas chromatography–mass spectrometry (GC–MS). These findings are expected to inform strain‐level food safety assessment and process control strategies for traditional lactic acid‐fermented vegetables in China.

## 2 Materials and Methods

### 2.1 Materials and Instruments

Fresh sauerkraut, fermentation tanks, and food‐grade salt (sodium chloride) were procured from local agricultural markets in Shanxi Province, China. Gas chromatography system (Model 7697A‐8860‐5977C; Agilent Technologies Inc.); electronic balance (Model FA224; Shanghai Sunny Hengping Scientific Instrument Co. Ltd.); laminar flow clean bench (Model VS‐1300 U; Shanghai Sujing Industrial Co. Ltd.); digital display blast drying oven (Model DHG‐9070A; Shanghai Jinghong Laboratory Equipment Co. Ltd.); induction cooker (Model C22‐RT22E01; Midea Group Co. Ltd).

### 2.2 Process Flow for Fermented Sauerkraut

Sauerkraut was produced through traditional spontaneous fermentation according to the following procedure: *Brassica rapa pekinensis*, selected for their thick leaf bases and minimal outer leaves, were first trimmed to remove residual roots and damaged leaves, followed by thorough washing with clean water. The prepared cabbage was then tightly layered in a fermentation vessel. Food‐grade salt was added at a concentration of 3% (w/w) relative to the total weight of the sauerkraut and evenly distributed between each layer. The vessel was sealed and maintained at a controlled temperature of approximately 10°C (with minor fluctuations of 2°C–3°C, monitored by a thermometer) for a fermentation period of 20 days, allowing for natural contraction and lactic acid fermentation.

### 2.3 Experimental Methods

#### 2.3.1 High‐Throughput Sequencing

Microbial community analysis was performed using high‐throughput sequencing. The V3‐V4 region of the bacterial 16S rRNA gene was amplified using universal primers (F: ACTCCTACGGGAGGCAGCA; R: GGACTACHVGGGTWTCTAAT). The fungal ITS1 region was amplified using primers (F: CTTGGTCATTTAGAGGAAGTAA; R: GCTGCGTTCTTCATCGATGC). Amplification and sequencing were conducted by Beijing Biomarker Technologies Corporation on an Illumina platform (Tang et al. 2025). Raw sequences were quality‐filtered and processed using QIIME2 (v2021.4).

#### 2.3.2 Determination of Flavor Components

##### 2.3.2.1 Headspace Conditions

Prior to headspace sampling, the fermented sauerkraut was ground into a paste using a mortar and pestle, from which a 2 g aliquot of the ground solid sample was weighed while 2 mL of the fermentation liquid was simultaneously collected; the solid and liquid samples were then separately transferred into 20 mL headspace vials, each supplemented with 2 mL of analytical‐grade saturated sodium chloride (NaCl) solution, before the vials were immediately sealed tightly with caps and prepared for subsequent analysis.

Headspace sampling was performed using an Agilent 7697A headspace sampler with the following parameters: oven temperature 110°C, loop temperature 120°C, transfer line temperature 130°C, and an equilibration time of 10 min. These conditions fall within the validated range reported for headspace‐GC analysis of fermented beverages (oven temperature 65°C–145°C, optimum approximately 102°C; equilibration time up to approximately 32 min), and the relatively short equilibration time used here was selected to minimize prolonged thermal exposure of the sample matrix and reduce the risk of thermally induced artifacts (Nurul Ain et al. 2023).

##### 2.3.2.2 GC–MS Detection Conditions

Gas Chromatography (GC) conditions: An HP‐5MS capillary column (30 m × 0.25 mm × 0.25 μm) was used. High‐purity helium (99.999%) served as the carrier gas at a constant flow rate of 1.0 mL/min. The injection was performed in split mode (split ratio 5:1) at 250°C, with the split flow maintained for 2 min. The oven temperature program was as follows: initial temperature held at 40°C for 3 min, increased to 150°C at a rate of 5°C/min, then raised to 250°C at 10°C/min, and finally held at 250°C for 5 min (Zhang et al. 2023; Li et al. 2025).

Mass Spectrometry (MS) conditions: The mass spectrometer was operated with an electron ionization (EI) source at 70 eV. The ion source temperature was set to 250°C. The data were acquired in full‐scan mode, with a mass scan range of m/z 35–400.

#### 2.3.3 Data Processing

The GC–MS data were analyzed using the Qualitative Analysis software (Agilent Technologies) integrated with the instrument. The obtained data were statistically processed and calculated using Microsoft Excel 2010 and SPSS software (version 23.0). Data visualization was performed using Origin 2021.

## 3 Results and Analysis

### 3.1 Analysis of Volatile Flavor Compounds in Fermented Sauerkraut

As shown in Table 1, Figures 1 and 2, a diverse profile of volatile flavor compounds was identified in the fermented sauerkraut. A total of 104 compounds were detected, which were categorized into 29 alcohols, 26 esters, 23 aldehydes, 7 ketones, 5 hydrocarbons, 4 organic sulfur compounds, 2 organic acids, and 8 other compounds. Notably, aldehydes were the most predominant group by relative content, accounting for 28.98%, with 5‐hydroxymethylfurfural (relative content: 12.34%) and furfural (relative content: 7.24%) as the two dominant contributors, imparting characteristic caramel and nutty aromas, respectively. This high abundance aligns with the characteristic volatile profile of Brassicaceae vegetables, indicating that aldehydes play a central role in defining the characteristic flavor of the fermented sauerkraut (Xie et al. 2025).

**TABLE 1:** Flavor substance composition table.

Serial number	Material category	Compound name	Molecular formula	CAS number	Relative content/%	Aroma description
1	Alcohols	1‐Hexanol	C_6_H_14_O	111‐27‐3	0.42	Aroma
2		3‐Methyl‐1‐butanol	C_5_H_12_O	123‐51‐3	18.28	Slightly sweet
3		Phenylethyl alcohol	C_8_H_10_O	60‐12‐8	1.15	Aliphatic alcohol notes
4		α‐Terpineol	C_10_H_18_O	98‐55‐5	2.15	Sweet floral
5		Dihydronopol	C_11_H_20_O	1000163‐46‐9	0.35	Sweetness
6		Cis‐p‐Mentha‐1(7)‐en‐2‐ol	C_10_H_16_O	22626‐43‐3	0.01	Lavender, pine or camphor scent
7		Longipinanol	C_15_H_26_O	39703‐23‐6	0.11	Rose fragrance
8		E‐Sesquilavandulol	C_15_H_26_O	120707‐27‐9	0.07	Fruity
9		α‐Acorenol	C_15_H_26_O	28296‐85‐7	0.11	Fruity
10		isoledene	C_15_H_24_	95910‐36‐4	0.16	Fruity
11		β‐Nootkatol	C_15_H_24_O	50763‐67‐2	0.03	Lavender, pine or camphor‐like scents
12		γ‐Muurolene	C_15_H_24_	30021‐74‐0	0.21	Melon fragrance
13		β‐Bisabolenol	C_15_H_24_O	147126‐90‐7	0.02	Waxy aroma
14		α‐Farnesene	C_15_H_24_	502‐61‐4	0.03	Camphor‐like scent
15		1‐Octyn‐3‐ol	C_8_H_14_O	818‐72‐4	0.2	Fruity and floral notes
16		1,3‐Butanediol	C_4_H_10_O_2_	107‐88‐0	0.02	Cool fragrance
17		2,3‐Butanediol	C_4_H_10_O_2_	513‐85‐9	1.75	Herbal and woody notes
18		3‐Octyn‐1‐ol	C_8_H_14_O	14916‐80‐4	0.04	Grassy scent
19		3‐Decyn‐2‐ol	C_10_H_18_O	69668‐93‐5	0.05	Floral and fruity notes
20		6‐Methyl‐2‐heptanol	C_8_H_18_O	4730‐22‐7	0.06	Fruity and floral notes
21		6‐Methyl‐5‐hepten‐2‐ol	C_8_H_16_O	1569‐60‐4	0.02	Fruity and floral notes
22		5‐Methyl‐6‐hepten‐1‐ol	C_8_H_16_O	26799‐28‐0	0.05	Lavender, floral and fruity notes
23		3,7‐Dimethyl−6‐octen‐1‐yn‐3‐ol	C_10_H_16_O	29171‐20‐8	0.13	Woody and fruity notes
24		7‐Hydroxyfarnesene	C_15_H_24_O	1000374‐20‐4	0.03	Fruity and floral notes
25		Trans‐p‐Mentha‐1(7),8‐dien‐2‐ol	C_10_H_16_O	21391‐84‐4	0.08	Fruity and floral notes
26		1H‐Cycloprop[e]azulen‐4‐ol	C_15_H_26_O	552‐02‐3	0.12	Fruit fragrance, fresh grass scent
27		Bicyclo[3.1.0]hexa4‐Methyl−1‐(1‐methylethyl)bicyclo[3.1.0]hexan‐3‐ol	C_10_H_18_O	513‐23‐5	0.5	Floral notes, woody tones Fruit
28		6‐(3‐Methyl‐3‐cyclohexenyl)‐2‐methyl‐2,6‐heptadienol	C_15_H_24_O	18681‐09‐9	0.01	Fragrance, fresh grass scent Fruit
29		3,7,11‐Trimethyl‐6,10‐dodecadien‐1‐yn‐3‐ol	C_15_H_24_O	2387‐68‐0	0.05	Fragrance, wax scent
1	Esters	Methyl thiolacetate	C_3_H_6_OS	1534‐08‐3	3.14	Fruity
2		Isobornyl acetate	C_12_H_20_O_2_	77318‐47‐9	2.47	Pine fragrance
3		Acetic acid, (acetyloxy)	C_4_H_6_O_4_	13831‐30‐6	0.64	Slight acidity and fruity aroma
4		(S)‐Isopropyllactate	C_6_H_12_O_3_	63697‐00‐7	0.13	Fruity, estery sweetness
5		Carquejol acetate	C_12_H_16_O_2_	32151‐09‐0	0.23	Fruity
6		Dihydrocarvyl acetate	C_12_H_20_O_2_	20777‐49‐5	0.52	Rose, floral notes
7		Dimethyl ethylidenemalonate	C_7_H_10_O_4_	17041‐60‐0	1.44	Fruity
8		Linalyl acetate	C_12_H_20_O_2_	115‐95‐7	0.5	Floral, fruity
9		Ethenyl formate	C_3_H_4_O_2_	692‐45‐5	0.03	Fruity
10		6‐Heptenyl acetate	C_9_H_16_O_2_	5048‐30‐6	0.14	Fruity
11		(3E,6Z)‐Nonadienyl acetate	C_11_H_18_O_2_	211323‐05‐6	0.15	Grassy notes, fruity notes
12		Myrcenyl acetate	C_12_H_20_O_2_	1000149‐84‐5	0.26	Fruity notes
13		(+)‐cis‐Verbenol, acetate	C_12_H_18_O_2_	29135‐27‐1	0.66	Herbal notes, floral notes
14		delta‐Nonalactone	C_9_H_16_O_2_	3301‐94‐8	0.46	Nutty aromas
15		(E)‐Jasmonyl acetate	C_13_H_22_O_2_	1,000,557–09‐1	0.07	Jasmine notes
16		Perillyl isobutyrate	C_14_H_22_O_2_	1000429‐32‐1	0.18	Fruity notes, floral notes
17		Butyl myrtenoate	C_14_H_22_O_2_	62486‐91‐3	0.15	Fruity notes, floral notes
18		Oplopanonyl acetate	C_17_H_28_O_3_	132032‐86‐1	1.24	Fresh, woody
19		8‐Ketoylangenal	C_15_H_20_O_2_	136458‐94‐1	0.05	Fruity, grassy and faint floral notes
20		(2E,4E)‐Octa‐2,4‐dienyl acetate	C_10_H_16_O_2_	30361‐34‐3	0.39	Fruity
21		Dihydro‐5‐methylfuran‐2(3H)‐one	C_5_H_8_O_2_	108‐29‐2	0.05	Fruity and strawberry fragrance
22		2,3‐Dihydro‐3,5‐dihydroxy‐6‐methyl‐4H‐pyran‐4‐one	C_6_H_8_O_4_	28564‐83‐2	0.33	Fruity
23		Bicyclo[3.1.1]hept‐2‐en‐6‐ol, 2,7,7‐trimethyl‐, acetate, [1S‐(1.alpha.,5.alpha.,6.beta.)]	C_12_H_18_O_2_	50764‐55‐1	0.28	Cedarwood fragrance
24		(Z)‐Ethyl but‐2‐enoate	C_6_H_10_O_2_	6776‐19‐8	0.32	Fruity
25		Methyl 2,5,10‐undecatrienoate	C_12_H_18_O_2_	14261‐55‐3	0.11	Minty notes, fruity notes, floral notes
26		3,7,11‐Trimethyl‐8,10‐dodecadienyl acetate	C_17_H_30_O_2_	1000374‐10‐4	0.09	Fruity, grassy
1	Aldehydes	Furfural	C_5_H_4_O_2_	98‐01‐1	7.24	Nutty aromas
2		Hexanal	C_6_H_12_O	66‐25‐1	1.06	Grassy aroma
3		Octanal	C_8_H_16_O	124‐13‐0	0.16	Fruity notes
4		Pentanal	C_5_H_10_O	110‐62‐3	0.12	Fruity and nutty notes
5		Heptanal	C_7_H_14_O	111‐71‐7	0.62	Strong waxy, grassy and fruity notes
6		Tetradecanal	C_14_H_28_O	124‐25‐4	0.39	Subtle fruity and floral notes
7		Methyl glyoxal	C_3_H_4_O_2_	78‐98‐8	0.35	Pungent, acrid odor
8		3‐Hydroxybutanal	C_4_H_8_O_2_	107‐89‐1	0.54	Fruity aroma
9		(E)‐2‐Heptenal	C_7_H_12_O	18829‐55‐5	0.34	Fruity, nutty notes
10		(+)‐4‐Carene	C_10_H_16_	29050‐33‐7	0.09	Woody, pine resin
11		2‐Methylene‐4‐pentenal	C_6_H_8_O	17854‐46‐5	0.43	Grassy, floral, fruity aromas
12		3‐Caren‐10‐al	C_10_H_14_O	1000151‐86‐0	0.27	Pine scent
13		Butanal, 3‐methyl—	C_5_H_10_O	590‐86‐3	0.08	Fruity, nutty
14		(2E,4E)‐2,4‐Nonadienal	C_9_H_14_O	5910‐87‐2	0.65	Fatty, floral notes
15		Benzeneacetaldehyde	C_8_H_8_O	122‐78‐1	1.12	Almond, cherry fragrance
16		gamma‐Terpinene	C_10_H_16_	99‐85‐4	1.49	Lemon scent
17		Trans‐Pinocarvyl acetate	C_12_H_18_O_2_	1686‐15‐3	0.28	Lavender, vanilla fragrance
18		5‐Hydroxymethylfurfural	C_6_H_6_O_3_	67‐47‐0	12.34	Caramel aroma
19		Cis‐Chrysanthemyl alcohol	C_10_H_18_O	18383‐59‐0	0.05	Floral and fruity notes
20		6‐Methyl‐3‐cyclohexene‐1‐carboxaldehyde	C_8_H_12_O	1000132‐13‐3	0.05	Fruity notes
21		1‐Cyclohexene‐1‐acetaldehyde,alpha,2‐dimethyl	C_10_H_16_O	53155‐80‐9	0.16	Minty aroma
22		4‐Isopropyl‐1‐cyclohexene‐1‐carbaldehyde	C_10_H_16_O	21391‐98‐0	1.12	Herbal essential oil fragrance
23		2,6‐Dimethyl−10‐methylene−2,6,11‐dodecatrienal	C_15_H_22_O	60066‐88‐8	0.02	Grass, fruity
1	Ketones	D‐Verbenone	C_10_H_14_O	18309‐32‐5	0.14	Minty
2		Xanthoxylin	C_10_H_12_O_4_	90‐24‐4	0.23	Sweet, woody
3		5‐Hexen‐2‐one	C_6_H_10_O	109‐49‐9	0.34	Fruity
4		3‐Octen‐2‐one, (E)	C_8_H_14_O	18402‐82‐9	0.06	Fruity
5		2‐Cyclohexen‐1‐one,4‐(1‐methylethyl)—	C_9_H_14_O	500‐02‐7	0.45	Fruity
6		1‐(2‐Ethyl‐1,3‐dimethyl‐cyclopent‐2‐enyl)‐ethanone	C_11_H_18_O	1010185‐18‐1	0.21	Jasmine
7		Trans‐Octahydronaphthalen−1(2H)‐one	C_10_H_16_O	21370‐71‐8	0.92	Pine, grass and floral
1	Hydrocarbons	p‐Cymene	C_10_H_14_	99‐87‐6	0.17	Pine resin aroma
2		Eucalyptol	C_10_H_18_O	470‐82‐6	0.3	Minty, woody notes
3		Ledene oxide‐(II)	C_15_H_24_O	1000159‐36‐7	0.07	Woody, sweet notes
4		(E)‐4‐Tridecen‐6‐yne	C_13_H_22_	74744‐43‐7	0.19	Fruity, floral notes
5		2‐Ethenyl‐1,3,3‐trimethylcyclohexene	C_11_H_18_	5293‐90‐3	0.38	Herbal notes
1	Organic sulfur compounds	Disulfide, dimethyl	C_2_H_6_S_2_	624‐92‐0	6.63	sulfurous odor, akin to garlic or onion
2		Dimethyl trisulfide	C_2_H_6_S_3_	3658‐80‐8	0.5	Onion scent
3		1‐Butene, 4‐isothiocyanato	C_5_H_7_NS	3386‐97‐8	0.57	Mustard and garlic aroma
4		Benzene, (2‐isothiocyanatoethyl)	C_9_H_9_NS	2257‐09‐2	0.82	Pungent, mustard‐like and sulfurous flavor
1	Organic acids	Acetic acid	C_2_H_4_O_2_	64‐19‐7	4.28	Sourness
2		Propanoic acid, 2‐oxo—	C_3_H_4_O_3_	127‐17‐3	2.73	Sourness
1	Other categories	2(5H)‐Furanone	C_4_H_4_O_2_	497‐23‐4	1.6	Fruity, caramel notes
2		3‐Ethylphenol	C_8_H_10_O	620‐17‐7	0.06	Caramel notes
3		Neointermedeol	C_15_H_26_O	5945‐72‐2	0.09	Fruity, floral notes
4		1,1′‐Oxydioctane	C_16_H_34_O	629‐82‐3	0.26	—
5		5‐Methylfurfural	C_6_H_6_O_2_	620‐02‐0	3.53	Caramel, nutty notes
6		trans‐Linalool oxide (furanoid)	C_10_H_18_O_2_	34995‐77‐2	0.71	Floral, fruity notes
7		(6‐Methylpyrazin‐2‐yl) methanol	C_6_H_8_N_2_O	77164‐93‐3	0.41	Roasted nutty notes
8		Naphthalene, decahydro‐1,4‐dimethoxy‐,(1.alpha.,4.beta.,4a.alpha.,8a.alpha.)	C_12_H_22_O_2_	41766‐66‐9	1.4	Fruity, herbal notes

*Note:* ‘—’ indicates that no aroma description was found for this substance.

**FIGURE 1:**
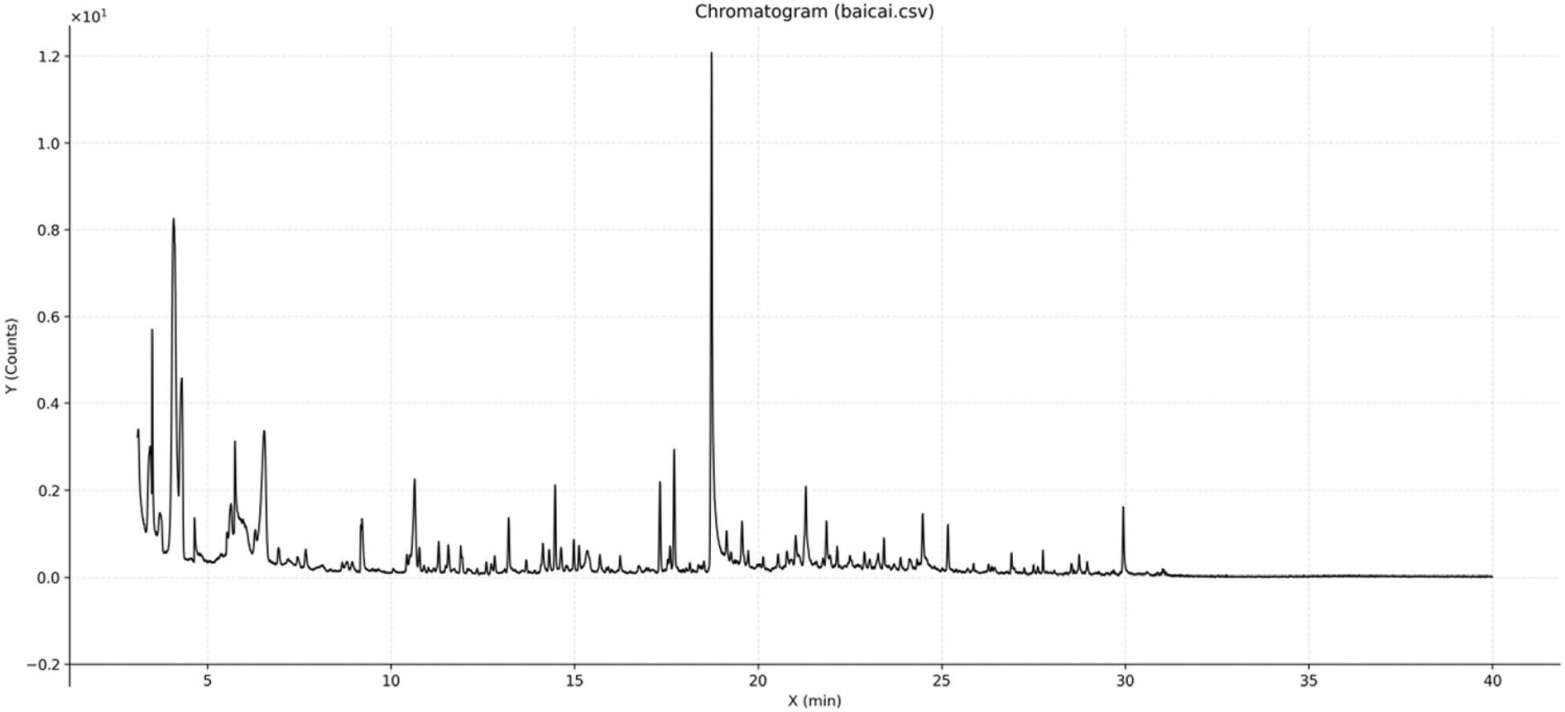
Gas chromatogram of sauerkraut.

**FIGURE 2:**
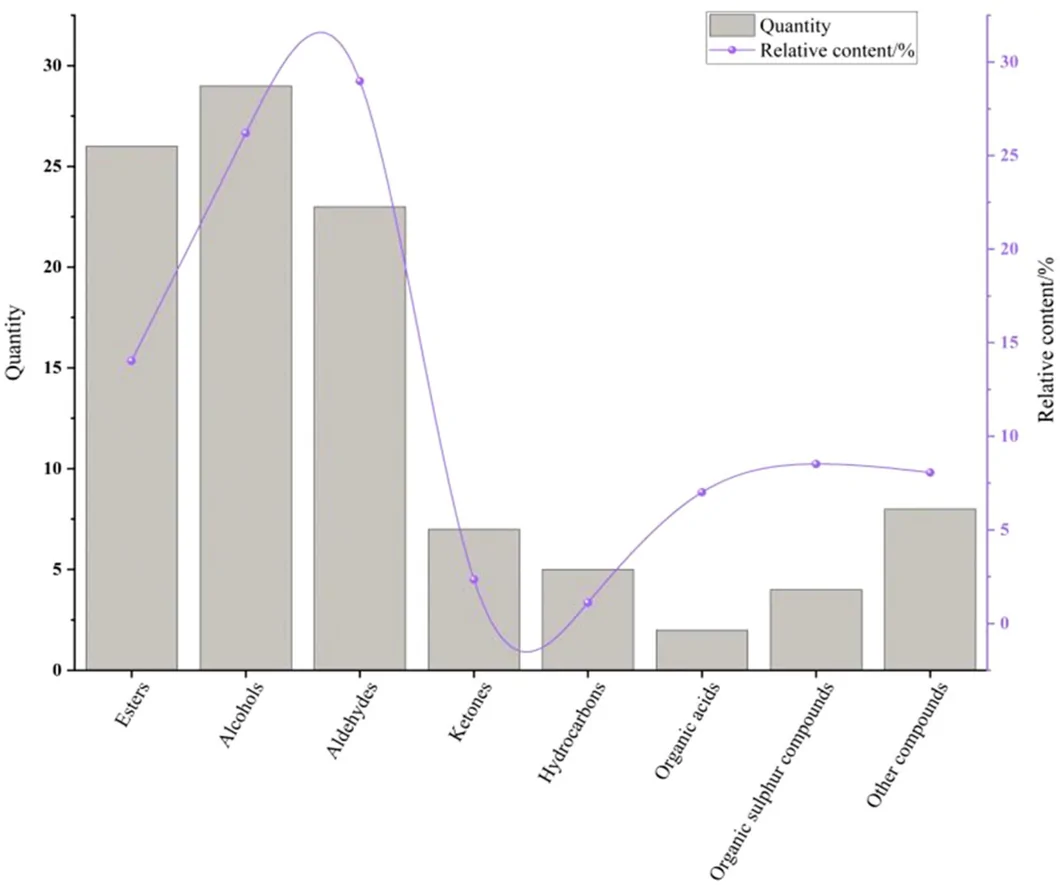
Classification of sauerkraut flavor substances.

Alcohols, as an indispensable group of flavor compounds, were also present in fermented sauerkraut at considerable levels. Among them, 3‐Methyl‐1‐butanol (relative content: 18.28%) exhibited a distinct aroma reminiscent of cider, accompanied by a spicy note (Gabur et al. 2024), contributing significantly to the overall flavor profile. 1‐Hexanol (relative content: 0.42%) imparted a fresh, fatty‐alcohol character, evoking scents of green fruits and cucumber (Chi et al. 2024), along with subtle waxy and floral nuances, thereby adding a fresh and delicate layer to the flavor complexity. α‐Terpineol (relative content: 2.15%), characterized by its lavender, pine, or camphor‐like aroma (Simona et al. 2020), played a role in harmonizing and enhancing the overall flavor complexity of the fermented sauerkraut.

Esters, renowned in food flavor for their characteristic fruity and sweet aromas, played a significant role in enhancing the flavor quality and hedonic perception of the fermented sauerkraut, despite their lower relative content compared to alcohols. For instance, methyl thiolacetate (relative content: 3.14%) contributed a distinct fruity note, while isobornyl acetate (relative content: 2.47%) imparted a woody, pine‐like scent (Li et al. 2016). These ester compounds acted synergistically with alcohols, aldehydes, and others, collectively forming the rich and harmonious flavor profile of the fermented sauerkraut.

### 3.2 Analysis of Microbial Diversity in Fermented Sauerkraut

#### 3.2.1 Alpha Diversity Analysis

Alpha diversity analysis reflects the species diversity within individual samples. The Chao1 and ACE indices estimate community richness (abundance) (Wu et al. 2025), while the Shannon and Simpson indices reflect community diversity (Liu, Li, et al. 2024). The results of the alpha diversity analysis for the fermented sauerkraut samples are presented in Table 2. As shown in the table, the sequence coverage for all samples reached 0.99, indicating a high coverage depth of the sample libraries. Compared to fungi, the bacterial communities exhibited higher Shannon indices and higher Simpson indices, suggesting a greater diversity of bacteria. In contrast, the higher Chao1 and ACE indices observed for fungi indicate a higher species richness of fungal communities (Sun et al. 2014).

**TABLE 2:** Alpha diversity indices of bacterial and fungal communities.

Sample ID	Shannon index	Simpson index	Chao1index	ACE index	Coverage
Bacteria	3.7135	0.8614	159	159	0.9999
Fungi	2.9569	0.6539	414.0909	387.8996	0.9996

#### 3.2.2 Dilution Curve Analysis

Rarefaction curves are an effective tool for assessing microbial community diversity and sequencing depth (Zhou et al. 2024). Bacterial rarefaction curves were based on 16S rRNA gene sequencing data; fungal curves were based on ITS1 region sequencing data (Figure 3). This indicates that the sequencing depth employed in this study was sufficient to capture the majority of bacterial and fungal species present in the fermented sauerkraut samples. The plateau observed in the curves suggests high coverage of the sequencing data, demonstrating that the data comprehensively reflect the diversity of the microbial communities (Luo et al. 2022).

**FIGURE 3:**
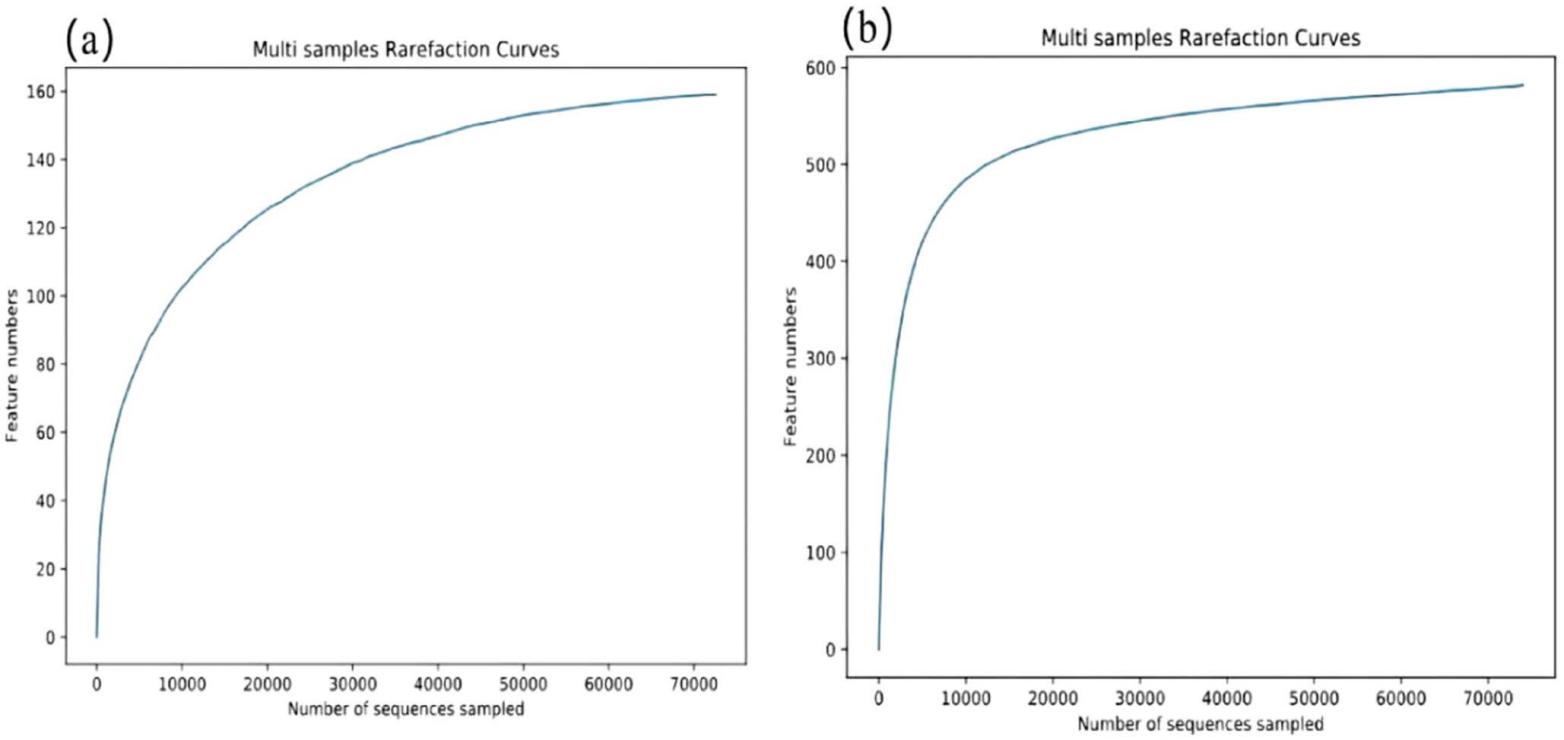
Alpha Index Dilution Curves: (a) (16S bacteria) and (b) (ITS fungi).

#### 3.2.3 Analysis of Bacterial Communities at the Genus and Phylum Levels

At the genus level, the predominant bacterial communities during the fermentation of sauerkraut included *Lactococcus*, *Enterococcus, Acinetobacter*, *Bacillus*, and *Leuconostoc*, as illustrated in Figure 4. Notably, *Lactococcus* and *Enterococcus*, as typical lactic acid bacteria (LAB), collectively accounted for a high abundance of > 50%, suggesting a close association with the formation of the core flavor profile. These LAB genera have been reported to contribute to acid production and may participate in flavor development through their metabolic activities (Chrysa et al. 2020). Subsequently, they enhance umami and flavor complexity by degrading proteins to generate free amino acids. Furthermore, they significantly influence the synthesis of volatile compounds, including alcohols, aldehydes, and esters (Table 1), imparting fruity and green notes to the sauerkraut. *Acinetobacter*, with a relative abundance of approximately 15%, might contribute to environmental adaptation and substrate utilization in the initial fermentation phase; however, its metabolites could be associated with off‐flavor risks, necessitating further investigation into its functional regulation. *Bacillus*, with a relatively low abundance, likely promotes the degradation of macromolecules by secreting proteases and lipases, indirectly affecting the accumulation of flavor precursors (Monareng et al. 2025).

**FIGURE 4:**
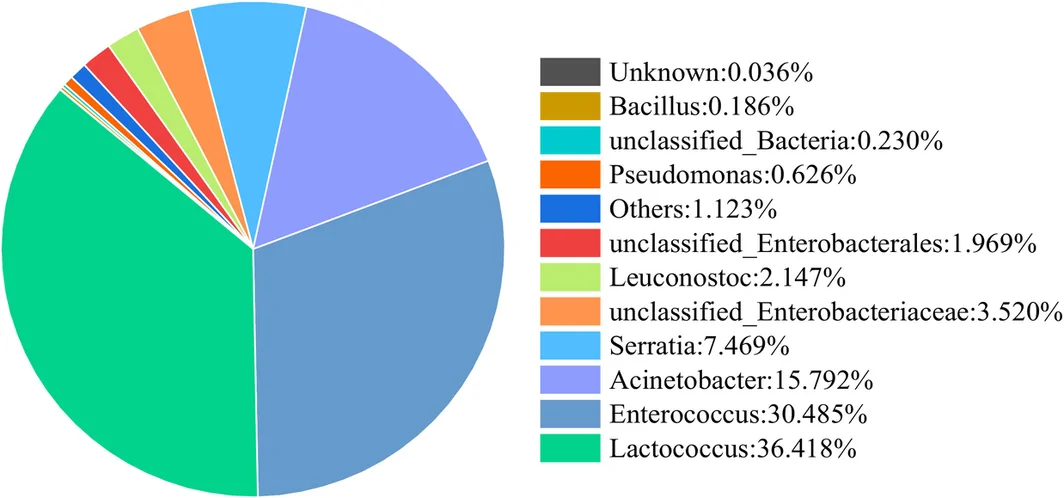
Distribution of bacteria at the genera level.

At the phylum level, the bacterial community during the fermentation of sauerkraut was predominantly composed of Firmicutes and Proteobacteria. As shown in Figure 5, the relative abundance of Firmicutes reached 69.37%, while that of Proteobacteria was 29.92%, collectively accounting for over 99% of the total bacterial community. This finding aligns with the typical characteristics of fermented sauerkraut, where lactic acid bacteria (LAB) within the Firmicutes phylum serve as the core functional microbiota (Liu, Wang, et al. 2024). These LAB are generally recognized as important contributors to organic acid production, including lactic acid and acetic acid, in fermented foods, which not only impart the characteristic sour taste but also inhibit the growth of undesirable microorganisms, thereby ensuring fermentation safety (Liu et al. 2022). Furthermore, other phyla, including Bacteroidota and Actinobacteria, were detected at minimal relative abundances (each < 1%). Despite their low abundance, they may contribute to specific metabolic pathways, such as protein degradation or the generation of flavor precursors, potentially enhancing the overall flavor complexity (Fu et al. 2021).

**FIGURE 5:**
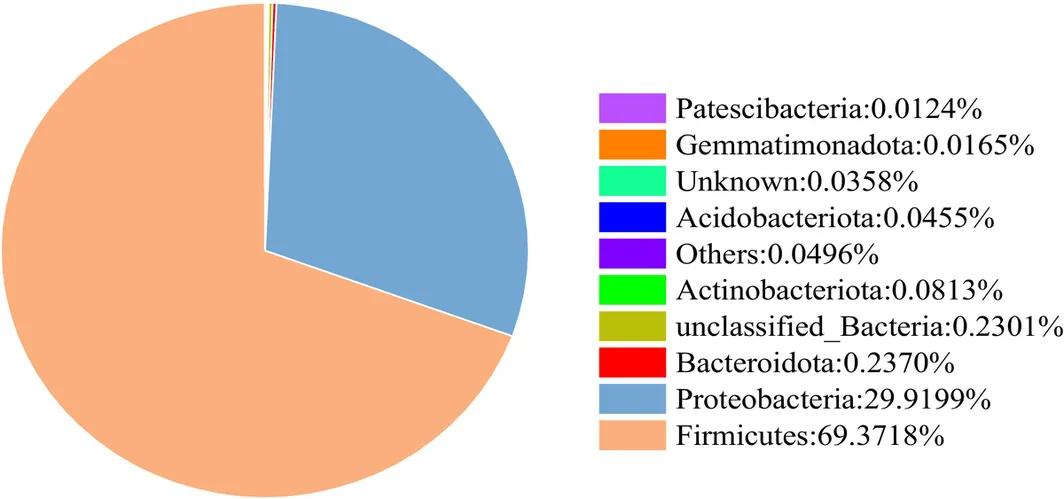
Distribution of bacteria at the phylum level.

#### 3.2.4 Analysis of Fungal Communities at Genus and Phylum Levels

As shown in Figure 6, at the genus level, *unclassified_Fungi* predominated with an absolute dominance of 53.79%, followed by *unclassified_Chytridiomycota* (3.11%) and *unclassified_Basidiomycota* (1.85%). Among the identifiable fungal genera, common environmental fungi such as *Alternaria* (0.37%), *Rhodotorula* (0.36%), and *Cladosporium* (0.32%) were detected, likely originating from the raw sauerkraut or the fermentation environment. Although present at low abundances, the metabolic activities of these fungi, including the secretion of cellulases or proteases, may contribute to substrate degradation and the formation of flavor precursors (Xu et al. 2024).

**FIGURE 6:**
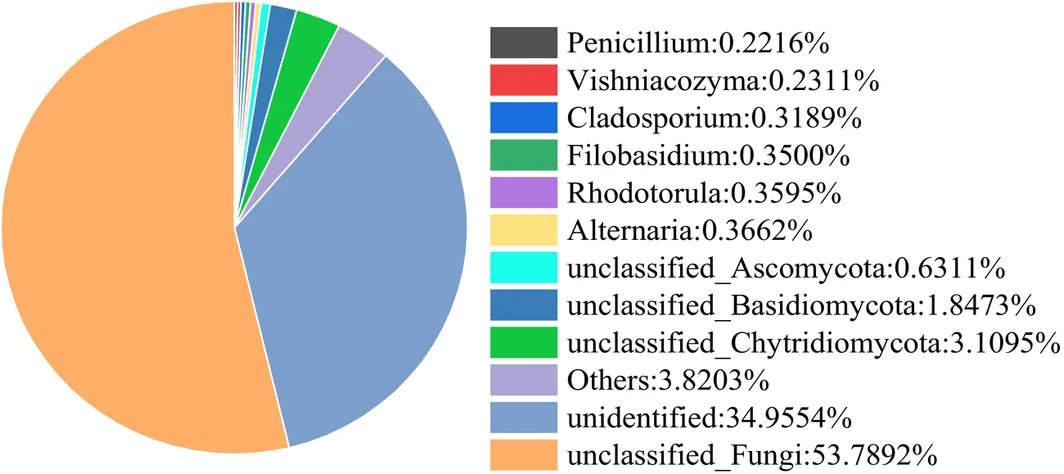
Distribution of fungi at the genus level.

At the phylum level, the fungal community during the fermentation of sauerkraut exhibited high diversity and was predominantly dominated by unclassified_Fungi and Chytridiomycota. As illustrated in Figure 7, the relative abundance of unclassified_Fungi was the highest at 53.79%, followed by Chytridiomycota at 33.49%. Other phyla were present at lower abundances: Ascomycota (4.10%), Basidiomycota (3.86%), and Rozellomycota (4.36%). The relatively low abundance of Ascomycota, which is commonly a functional fungal phylum in traditional fermented foods, may be attributed to the low‐temperature fermentation environment of the sauerkraut, which likely suppressed the growth of some mesophilic fungi. Furthermore, the detection of rare phyla such as Mortierellomycota and Glomeromycota (collectively < 1%) suggests the existence of complex ecological interactions within the fungal community of the fermentation system.

**FIGURE 7:**
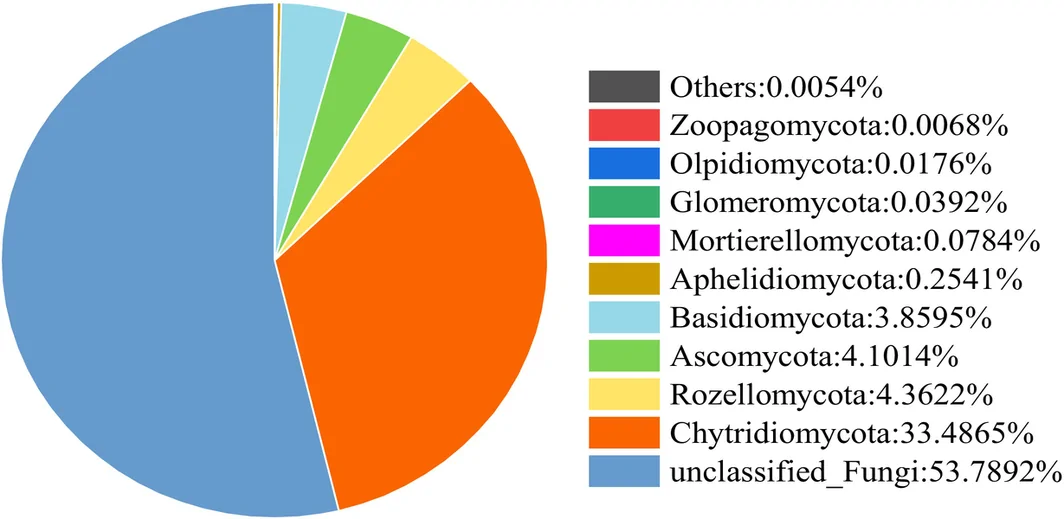
Distribution of fungi at the phylum level.

### 3.3 Analysis of the Correlation Between Microorganisms and Volatile Flavor Compounds

Figure 8 presents the correlation analysis between the microbial community and volatile flavor compounds during the fermentation of sauerkraut. The heatmap illustrates the strength of correlations between different microbial genera and volatile flavor compounds through a color code: red indicates a positive correlation, blue represents a negative correlation (Tian et al. 2023), and the color intensity corresponds to the magnitude of the correlation (Lu et al. 2024). As observed, dominant microbial genera exhibited significant correlations with various volatile flavor compounds. At the genus level, *Lactococcus* and *Enterococcus* showed strong positive correlations (dark red) with several acetate esters, suggesting a potential role in the formation of these flavor compounds. Similarly, *Acinetobacter* exhibited positive correlations with methyl thiolacetate, delta‐nonalactone, and several ester compounds, indicating its possible contribution to sulfur‐containing compound metabolism and lipid‐derived flavor transformation. In contrast, *Pseudomonas* and *Serratia* displayed negative correlations with several alcohols and esters, suggesting that their abundance variations may be associated with the reduced accumulation of certain volatile compounds. Furthermore, at the phylum level, Firmicutes showed positive correlations with multiple volatile flavor compounds, particularly alcohols and esters, highlighting their potential contribution to flavor development during fermentation. Conversely, Proteobacteria exhibited both positive and negative correlations with different volatile compounds, indicating their complex associations with flavor formation.

**FIGURE 8:**
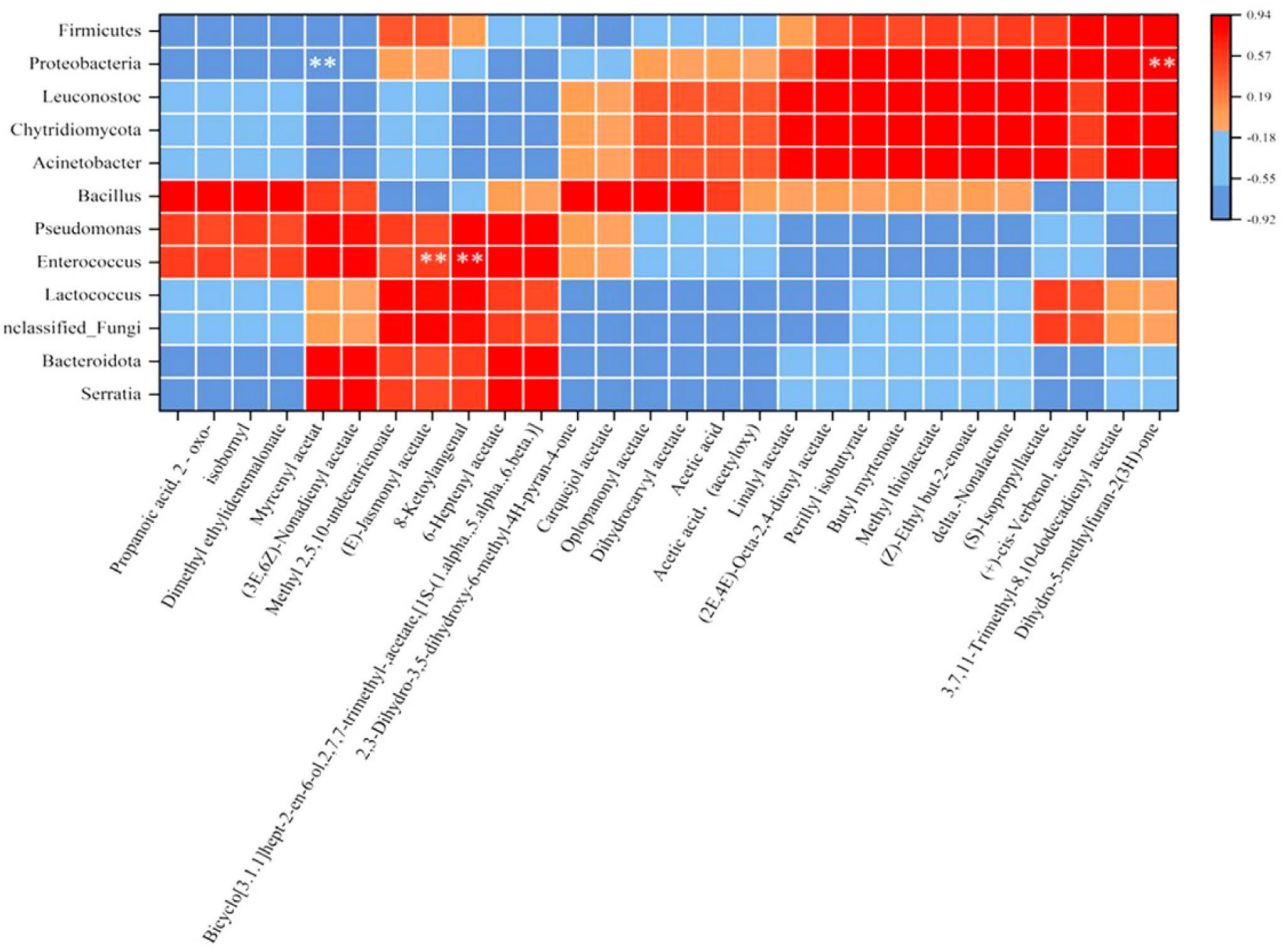
Heat map of correlation between microorganisms and volatile flavor substances.

These results elucidate the influences of specific bacterial genera on the formation of volatile flavor compounds in sauerkraut. *Lactococcus* and *Enterococcus*, as the dominant microorganisms during fermentation, may be closely associated with shaping the flavor profile of sauerkraut through their metabolic activities. These findings provide significant insights into the complex interplay between microbial dynamics and flavor development in fermentation processes. Furthermore, the data also reveal several negative correlations, indicating that these genera may play a role in suppressing the production or transformation of certain flavor compounds.

In summary, the correlation analysis between microorganisms and volatile flavor compounds demonstrates that the microbial community structure during sauerkraut fermentation significantly influences the formation of flavor substances. These correlations highlight *Lactococcus* and *Enterococcus* as candidate targets for future starter‐culture screening aimed at improving flavor consistency in sauerkraut fermentation.

## 4 Discussion

In this study, the correlation between microbial community structure and flavor compounds in sauerkraut was investigated using high‐throughput sequencing and GC–MS. A total of 104 volatile flavor compounds were identified by GC–MS, among which aldehydes accounted for 28.98% of the relative content, representing a major contributor to the characteristic flavor profile. This high proportion of aldehydes is consistent with the typical volatile profile of Brassicaceae vegetables. Regarding bacterial communities, Firmicutes and Proteobacteria were the dominant phyla, while *Enterococcus* and *Lactococcus* were the dominant genera. Regarding fungal communities, a high proportion of unclassified sequences was observed, which may reflect limitations in current fungal reference databases or the presence of understudied species in the fermentation environment. Correlation analysis revealed significant positive associations between *Enterococcus* and *Lactococcus* and several acetate‐derived esters, suggesting a potential contribution to the accumulation of fruity aroma compounds, pending further metabolic validation.

The dominance of *Enterococcus* and *Lactococcus* observed in this study is broadly consistent with recent findings in other traditionally fermented sauerkraut systems in China, where *Lactococcus* and *Lactobacillus* were similarly identified as genera significantly and positively correlated with volatile flavor metabolites (Wang et al. 2023). However, the specific genus‐level composition observed here—particularly the high relative abundance of *Enterococcus*—differed from that study, in which *Lactobacillus* was the most dominant genus; this discrepancy may reflect the low‐temperature and high‐salt fermentation conditions used in the present study, as well as differences in raw material and regional microbial inoculum sources. It is also worth noting that, while *Enterococcus* contributes functionally to flavor formation in many fermented foods, certain strains within this genus are recognized as opportunistic pathogens and carriers of antibiotic resistance genes (Hanchi et al. 2018). As strain‐level identification and safety screening were not performed in this study, future work should incorporate genomic characterization of the dominant *Enterococcus* population to confirm its food‐safety status alongside its functional contribution to flavor.

Day 20 was selected as the sampling point in this study because it represents a commonly used endpoint at which sauerkraut fermentation is considered to approach microbiological maturity under low‐temperature fermentation conditions under the low‐temperature (approximately 10°C) conditions used here, consistent with common industrial practice for this product. However, we acknowledge that analyzing only this single, mature‐stage time point does not capture the dynamic succession of microbial communities and flavor compounds throughout the fermentation process and precludes tracking how specific taxa emerge or decline over time. In addition, this study did not monitor physicochemical parameters such as pH, titratable acidity, salt dynamics, or sugar consumption, which are known to shape microbial metabolism and, consequently, flavor compound formation. Together, these limitations restrict our ability to infer causal relationships between microbial taxa and flavor compounds and to mechanistically interpret the observed correlations. Future studies should incorporate multi‐timepoint sampling across the full fermentation period, together with concurrent physicochemical monitoring, to more comprehensively elucidate the dynamic interactions between microbiota, environmental conditions, and flavor development.

## 5 Conclusions

In this study, the correlation between microbial community structure and flavor compounds in sauerkraut was investigated using high‐throughput sequencing and GC–MS. A total of 104 volatile flavor compounds were identified, dominated by aldehydes (28.98%), alcohols (3‐methyl‐1‐butanol, 18.28%), and esters (methyl thiolacetate, 3.14%), while organic acids such as acetic acid (4.28%) contributed to the characteristic sourness of fermented sauerkraut. Firmicutes and Proteobacteria were the dominant phyla, with *Enterococcus* and *Lactococcus* as dominant genera potentially associated with flavor development. Correlation analysis revealed significant positive associations between these two genera and several acetate‐derived esters, suggesting their potential contribution to the accumulation of fruity aroma compounds; confirming a causal role will require further metabolic validation. Overall, these results offer a working framework linking sauerkraut microbiota to flavor compound accumulation, which can help prioritize target taxa for future mechanistic and applied research. Nonetheless, as this study was based on a single fermentation time point without concurrent physicochemical monitoring, the observed relationships should be regarded as preliminary and correlational. Future studies incorporating multi‐timepoint, dynamic sampling together with physicochemical monitoring and metabolic validation are warranted to confirm these associations and further elucidate the underlying mechanisms.

## Author Contributions

**Xiangcheng Lu:** investigation, formal analysis, validation, methodology. **Shiyu Guo:** conceptualization, investigation, formal analysis, validation. **Hongmei Zhao:** formal analysis, methodology. **Yanjun Yang:** formal analysis, visualization, validation. **Xiaoli Ma:** validation, formal analysis, visualization. **Shengli Shi:** validation, formal analysis, visualization. **Bianfang Hu:** resources, supervision, writing – review and editing. **Aiguo Luo:** conceptualization, formal analysis, funding acquisition, project administration, resources, writing – review and editing.

## Funding

This study was supported by the “Fund Program for the Scientific Activities of Selected Returned Overseas Professionals in Shanxi Province” (20250038).

## Conflicts of Interest

The authors declare no conflicts of interest.

## Data Availability

The data that support the findings of this study are available on request from the corresponding author. The data are not publicly available due to privacy or ethics restrictions.
